# A dataset of microstructure features of electro-hydrodynamic assisted 5-fluorouracil-grafted alginate microbeads and physicochemical properties for effective colon targeted carriers drug delivery

**DOI:** 10.1016/j.dib.2024.110202

**Published:** 2024-02-20

**Authors:** Abul Kalam Azad, Wan Mohd Azizi Wan Sulaiman, Hassan Almoustafa, Mohamad Dayoob, Vinoth Kumarasamy, Vetriselvan Subramaniyan, Jamilah M. Alshehri, Azmat Ali Khan

**Affiliations:** a University College of MAIWP International, 68100 Batu Caves, Kuala Lumpur, Malaysia; b Department of Pharmacolgy, Faculty of Medicine, University of Malaya 50603 Kuala-Lumpur, Malaysia; c Faculty of Pharmacy, MAHSA University, 42610 Jenjarom, Selangor, Malaysia; d Department of Parasitology and Medical Entomology, Faculty of Medicine, Universiti Kebangsaan Malaysia, Jalan Yaacob Latif, 56000 Cheras, Kuala Lumpur, Malaysia; e Pharmacology Unit, Jeffrey Cheah School of Medicine and Health Sciences, Monash University, Jalan Lagoon Selatan, Bandar Sunway, 47500 Selangor Darul Ehsan, Malaysia; f Pharmaceutical Biotechnology Laboratory, Department of Pharmaceutical Chemistry, College of Pharmacy, King Saudi University, Riyadh 11451, Saudi Arabia

**Keywords:** 5-fluorouracil, Electrospray, Microbeads, pH sensitive delivery, Sodium alginate

## Abstract

5-Fluorouracil (5-FU) has been the primary drug used in chemotherapy for colorectal carcinoma, and localizing the drug would be effective in avoiding its side effects and improving therapeutic outcomes. One approach to achieve this is by encapsulating the drug in microbeads. Alginate microbeads, in particular, exhibit promising pH-sensitive properties, making them an attractive option for colon targeting. Thus, the main aim of this study is to formulate and characterize 5-FU-encapsulated alginate microbeads as a pH-sensitive drug delivery system for controlled release in the gastrointestinal tract. In this study, the alginate microbeads encapsulating 5-FU was manufactured using electrospray methods. This method offers the advantages of promoting the formulation of uniformly small-sized microbeads with improved performance in terms of swelling and diffusion rates. The size and shape of the 5-FU microbeads are 394.23 ± 3.077 µm and have a spherical factor of 0.026 ± 0.022, respectively, which are considered acceptable and indicative of a spherical shape. The microbeads' encapsulation efficiency was found to be 69.65 ± 0.18%, which is considered high in comparison to other literature. The attenuated total reflectance - Fourier transform infrared spectroscopy (ATR-FTIR) data confirmed the complexation of sodium alginate with calcium ions, along with the encapsulation of 5-FU in the microbeads matrix. The 5-FU microbeads displayed pH-dependent swelling, exhibiting less swelling in simulated gastric fluid (SGF) than in simulated intestinal fluid (SIF). Additionally, the release of 5-FU from the microbeads is pH-dependent, with the cumulative percentage drug release being higher in simulated intestinal fluid than in SGF. The data indicate that the 5-FU microbeads can be utilized for the delivery of 5-FU in colon-targeted therapy, potentially leading to improved tumor treatment.

Specifications Table

**Table:** 

Subject	Pharmaceutics
Specific subject area	Targeted Drug Delivery Systems
Data format	Raw Analyzed
Type of data	Table Figure SEM image
Data collection	Data were collected as a part of final year project. A digital camera was used to capture the image of the dried microbeads. As for the shape and size, a Sigma ScanPro 5 image analyser was used for that purpose. Scanning Electron Microscope (SEM) for observation of the morphology and structure of the microbeads. Encapsulation efficiency was measured by UV-spectrophotometer. Particle size distribution data was analyzed and collected using dynamic light scattering (DLS).
Data source location	This experiment was carried out at the Faculty of Medicine, University Malaya located in 50603 Kuala Lumpur, Federal Territory of Kuala Lumpur and Maker4IR, Bandar Indera Mahkota, 25200 Kuantan, Pahang, Malaysia.
Data accessibility	Repository name: Mendeley data Data identification number: 10.17632/hgtphykjnb.1 Direct URL to data: https://data.mendeley.com/datasets/hgtphykjnb/1
Related research article	Azad, A. K., Al-Mahmood, S. M. A., Kennedy, J. F., Chatterjee, B., & Bera, H. (2021). Electro-hydrodynamic assisted synthesis of lecithin-stabilized peppermint oil-loaded alginate microbeads for intestinal drug delivery. International journal of biological macromolecules, 185, 861–875. https://doi.org/10.1016/j.ijbiomac.2021.07.019

## 1 Value of the Data

• Datasets explain about formulation of 5-FU encapsulated alginate microbeads for controlled drug release in the GI tract using electrospray technique.
• Datasets describe characterisation of the physical properties 5-FU loaded alginate microbeads
• Datasets explain the swelling and drug release profile of the microbeads using in vitro testing.
• The data indicates that electrohydrodynamic atomization is an effective method for preparing alginate-based microbeads, enabling pH-responsive targeted delivery of 5-FU.
• This development represents a significant advancement in drug delivery technology for colon cancer treatment, offering controlled release, drug protection, and precise targeting within the colon's environment. Also, this data represents a step forward in enhancing the effectiveness and minimizing side effects of 5-FU treatment for colon cancer.
• The use of electrohydrodynamic atomization for preparing alginate-based microbeads is an innovative method. This can inspire other researchers to explore similar techniques for their own drug delivery systems. Furthermore, the demonstration of pH-responsive targeted drug delivery of 5-FU provides valuable insights. This knowledge can be used as a foundation for designing and optimizing drug delivery systems for various types of cancer or other diseases.

## 2 Data Description

### 2.1 Particle size distribution and zeta potential of 5–fluorouracil emulsion

**Fig. 1:**
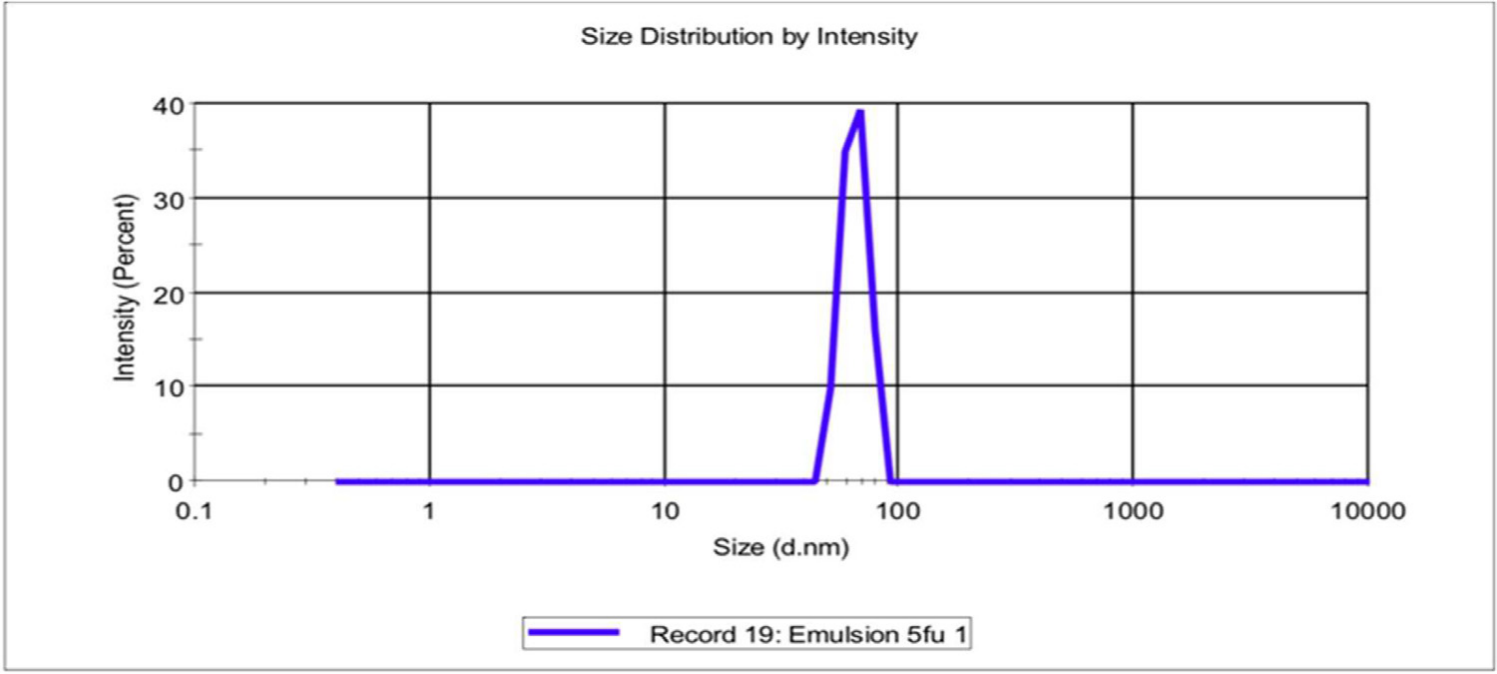
The graph of particle size distribution of 5-FU-ALG emulsion by intensity.

This data has deposited in Mendeley data with folder name as “Dataset of microstructure features of electro-hydrodynamic assisted alginate microbeads” and file name “Physicochemical properties of 5-FU-grafted alginate microbeads”. Here, Fig. 1 shows the particle size distribution and polydispersity index of the 5-fluorouracil emulsion. Since the PDI is more than 0.3, this indicates that the emulsion does not have a homogeneous particle size distribution, as suggested. PDI values less than 0.3 are considered homogeneous [1].

**Fig. 2:**
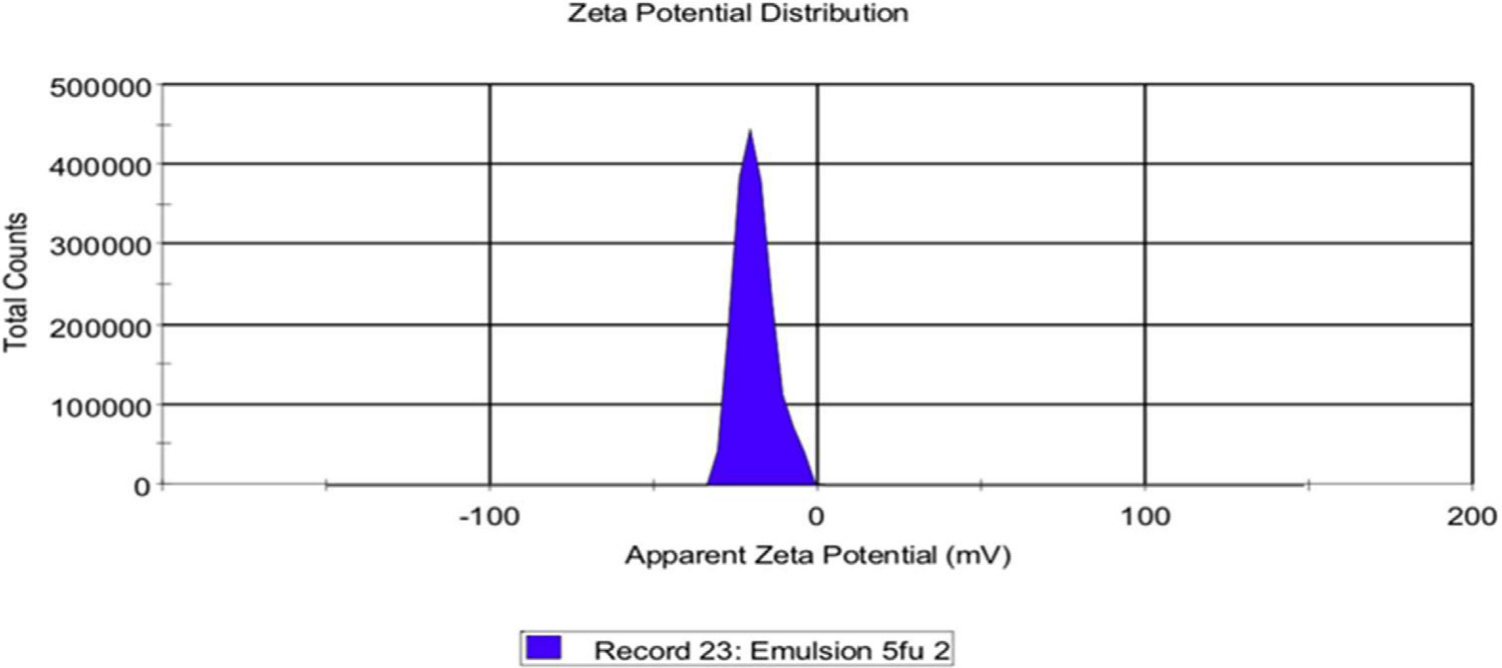
The graph of zeta potential of 5-FU-ALG emulsion.

Fig. 2 illustrates the zeta potential of the 5-fluorouracil emulsion. In this finding, the zeta potential was −19.2 ± 5.80 mV. According to Azad et al. (2020), zeta potential plays an important role in the stability of the colloidal structure of the emulsion [2].

### 2.2 Shape and size of microbeads

**Fig. 3:**
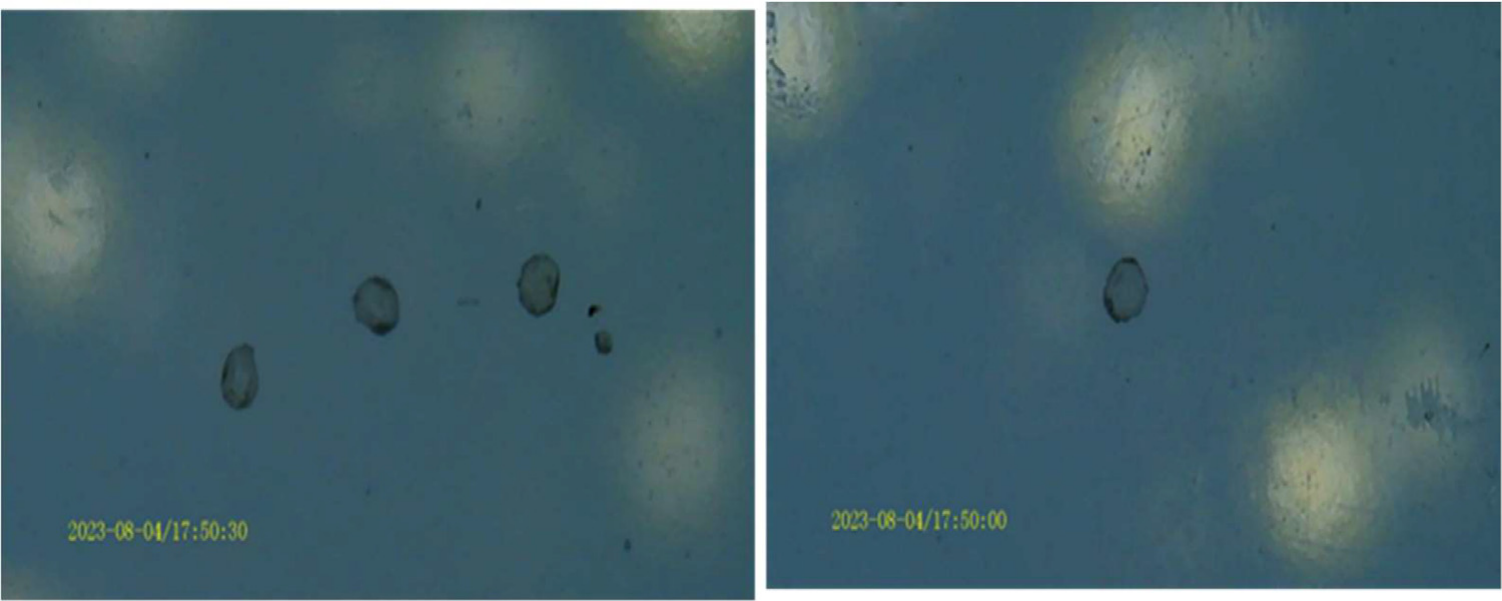
The image of 5-FU microbeads under digital microscope.

The shape of the 5-FU microbeads under the digital microscope is illustrated in Fig. 3. The produced 5-FU microbeads have a size range of 394.233 ± 3.077 µm, with spherical factors of 0.024 ± 0.022 as shown in Table 1. Since the 5-FU microbeads produced are under 1000 µm, they theoretically fall into the category of microparticles. Additionally, the spherical factors of the 5-FU microbeads produced are less than 0.05. Therefore, they can be considered to have a spherical shape [2].

**Table 1:** Size and spherical factor of 5-FU microbeads.

Sample	Size of 5-FU microbeads (µm)	Spherical factor of 5-FU microbeads
1	393.8	0.026
2	390.7	0.002
3	398.2	0.046
Mean	394.233 ± 3.077 µm	0.024 ± 0.022

### 2.3 Encapsulation efficiency

The encapsulation efficiency of the produced 5-FU microbeads was 69.65 ± 0.18%. Gowda et al. (2020) reported an encapsulation efficiency range of 58.3–74.4% with 5-FU [3], while Ramana and Krishna (2011) reported encapsulation efficacy ranging from 63.88 to 69.84% [4]. Thus, the encapsulation in this research can be considered moderately high compared to those previous findings. This may be attributed to the high voltage applied during the encapsulation process. This higher voltage can increase the cross-linking affinity towards the CaCl2 solution [2]. Consequently, it leads to the entrapment of the 5-FU and prevention of its release from the alginate matrix through ionic cross-linking, which are present in high numbers [2].

### 2.4 Microbeads weight uniformity

In order to ensure the production of a high-quality product, it is crucial to achieve uniformity in the weight of the microbeads. This factor significantly impacts the overall product quality. As shown in Table 2, the produced 5-FU microbeads have a mean weight of 0.93 mg for 30 microbeads, indicating an average weight of 0.031 mg per individual 5-FU microbead. Additionally, the coefficient of weight variation is reported to be 5.37%, a figure very close to the specifications outlined in the USP Pharmacopeia. This data indicates a high level of uniformity in the microbead weights, demonstrating consistent encapsulation of the 5-FU within the alginate microbeads using the electrospray technique

**Table 2:** Weight uniformity of the 5-FU microbeads.

Sample	Weight of 30 5-FU microbeads (mg)	Weight of a 5-FU microbeads (mg)	Coefficient of Weight Variation (%) = (SD/mean) × 100
1	0.90	0.03	5.37
2	0.90	0.03	
3	1.00	0.033	
Mean	0.90 ± 0.050	0.93 ± 0.001	

### 2.5 Scanning electron microscopy

**Fig. 4:**
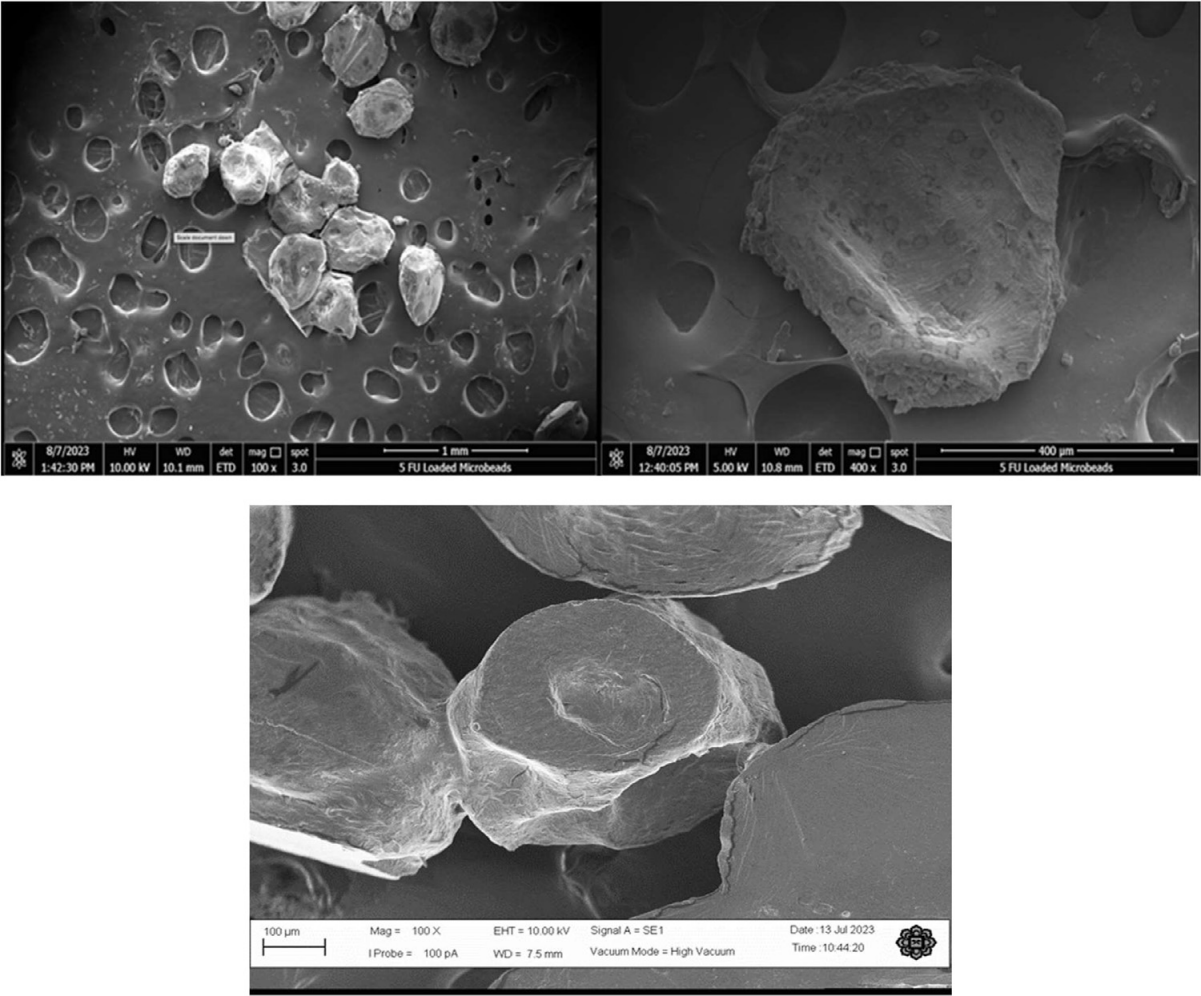
The scanning electron microscope image of the 5-FU microbeads (Left), cross section of 5-FU microbeads (Right) and without 5-FU leaded/blank microbeads (bottom).

Under the scanning electron microscope (SEM), the image of the 5-FU-loaded alginate microbeads reveals numerous pores and channels on their external surface (Fig. 4). This phenomenon may be attributed to the space left by water molecules during the evaporation process of drying [2]. Meanwhile, the spots observed on the internal surface of the cut microbead are likely a result of the encapsulation of the 5-FU emulsion within the alginate matrix.

### 2.6 Attenuated total reflectance-Fourier transform infrared spectroscopy (ATR-FTIR)

**Fig. 5:**
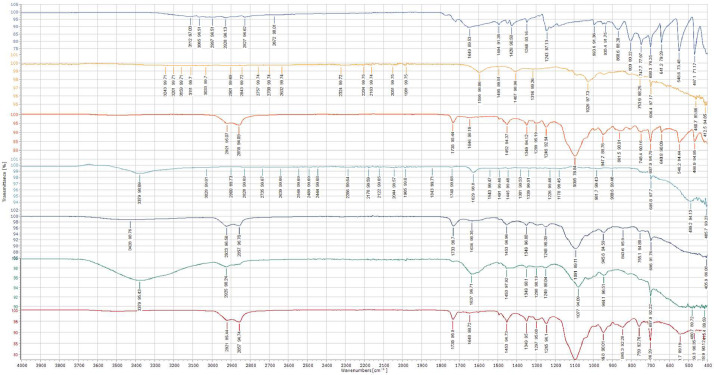
ATR-FTIR spectra of (a) 5-FU, (b) sodium alginate, (c) Tween 80, (d) physical mixture, (e) calcium chloride, (f) 5-FU loaded microbeads (g) blank microbeads.

Fig. 5 shows the ATR-FTIR spectra of the 5-FU, sodium alginate, CaCl_2_, 5-FU loaded microbeads, blank microbeads and the physical mixture. For 5-FU, the peaks were noticeable in 3200 cm^−1^ for N—H stretching vibrations, 3065 cm^−1^ for C—H stretching vibrations, 2928–2827 cm^−1^ for CH_2_, 1649 cm^−1^ for C=C stretching vibration, 1510 cm^−1^, 1494 cm^−1^ and 1428 cm^−1^ indicating N—H plane bending vibration, 1243 cm^−1^ showing ring stretching vibrations, 993.6 cm^−1^ and 935.4 cm^−1^ showing N—H and C—H wagging vibrations respectively. There are also C-F stretching vibrations at about 803 cm^−1^ and pyrimidine ring vibration at about 747.7 cm^−1^ which were similar to the findings reported in [3].

The peak in the sodium alginate spectra at 1595 cm^−1^ and 1407 cm^−1^ corresponds to characteristic bands, with 1595 cm^−1^ representing the C=O group and 1407 cm^−1^ indicating the C—C group stretching vibration. It was reported that the band between 1600 and 1405 cm^−1^ corresponds to the asymmetric and symmetric -COOH groups' stretching bands. Additionally, the bands observed at 1316 to 1025 cm^−1^ in the sodium alginate spectra indicate the presence of the C—H group. Tween 80 exhibits bands at 2921–2855 cm^−1^, signifying the presence of the -CH2 group, while the band at 1735 cm^−1^ indicates the C=O ester group. The peak at 1646 cm^−1^ represents HOH bending, and the peaks in the range of 1316–1026 cm^−1^ indicate asymmetric and symmetric stretching bands [5]. Regarding CaCl2, the broad band at 3379 cm^−1^ indicates the presence of OH stretching vibration, most likely due to residual water in the powder. Meanwhile, the peak at 1613 cm^−1^ signifies the Ca-Cl symmetrical stretching bond [2].

The physical mixture of Tween 80, alginate and 5-FU spectra seems to be the overlay of the respective individual spectra in which the peaks of the ingredient's spectra appear in their specific locations. For instance, the peaks of Tween 80 can be seen in the physical mixture spectra of 2921 cm^−1^, 2858 cm^−1^, 1735 cm^−1^ and 1095 cm^−1^. Similar observations were found for the 5-FU with peaks present on 1243 cm^-1^. In the 5-FU loaded microbeads spectra, most of the 5-FU peaks were not seen. This means that the 5-FU has been encapsulated in the microbeads and there is no 5-FU present on the surface of the microbeads [2].

Moreover, there are some changes in the peak of sodium alginate spectra when it is shown in the blank and 5-FU loaded microbeads spectra. For example, the 3243 cm^−1^ peak that indicates the presence of the carbonyl group has shifted from 3243 cm^−1^ to a higher value in microbeads which are 3379 cm^−1^ in blank microbeads and 3428 cm^-1^ in 5-FU loaded microbeads. This indicates the complexation of the sodium alginate with the calcium ions. As a confirmation of the complex formation, the absence of 1613 cm^−1^ in the blank and 5-FU loaded microbeads spectra that indicates the absence of Ca-Cl band prove the complexation of calcium with sodium alginate.

### 2.7 Swelling characteristics

**Fig. 6:**
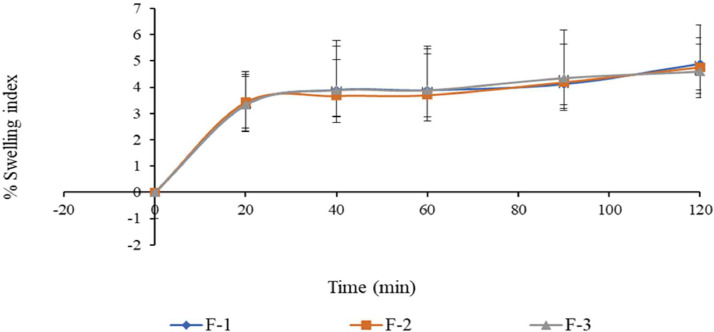
Percentage swelling index of 5-FU microbeads in HCl (Mean 土 SD, *n* = 3).

**Fig. 7:**
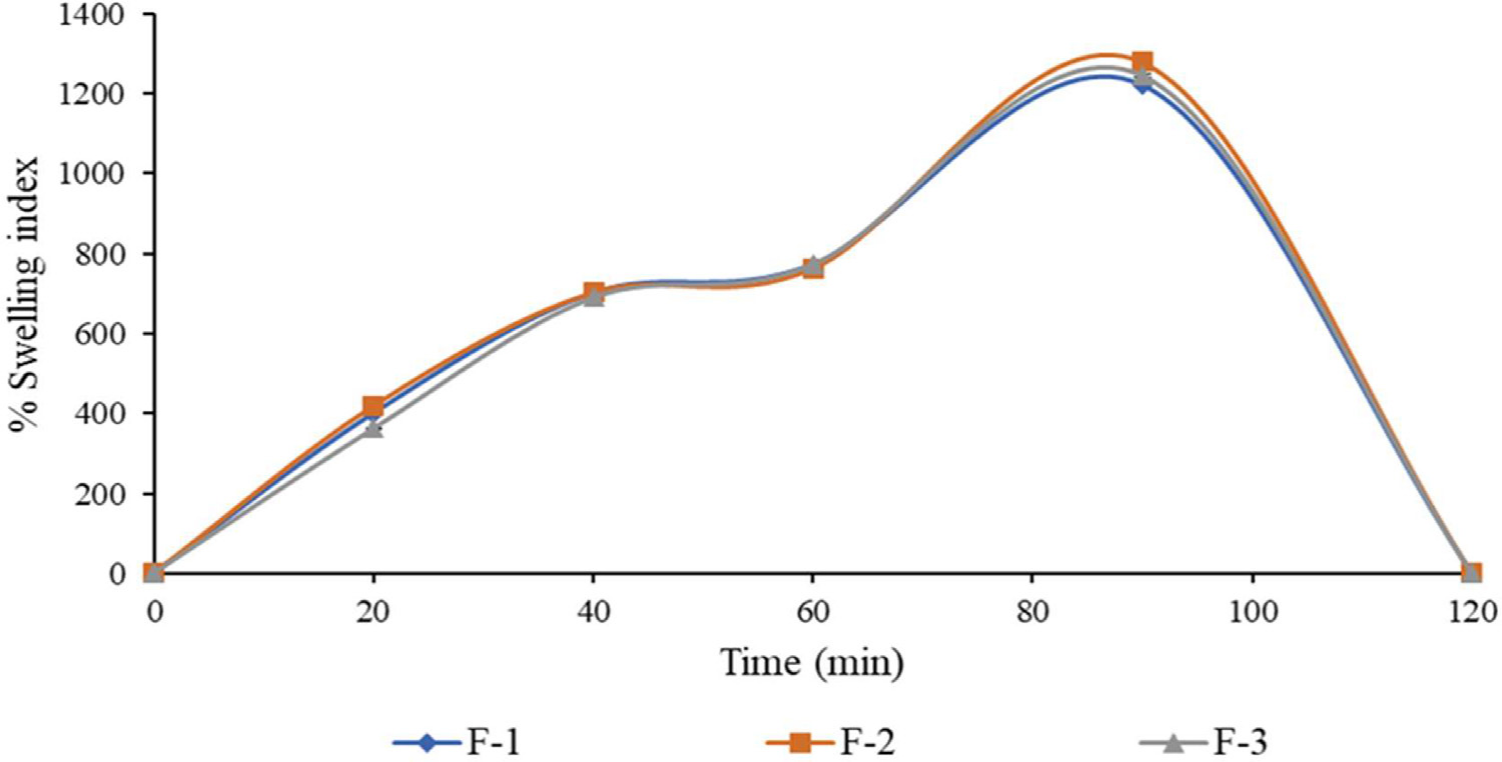
Percentage swelling index of 5-FU microbeads in phosphate buffer (Mean 土 SD, *n* = 3).

The 5-FU microbeads were tested in simulated gastric fluid (SGF) with a pH of 1.2 and simulated intestinal fluid (SIF) with a pH of 6.8, as depicted in Figs. 6 and 7.

All 5-FU microbeads exhibited a low percentage swelling index in the SGF, whereas in SIF, they displayed a high percentage swelling index. This indicates that the 5-FU alginate microbeads possess pH-sensitive swelling properties. For example, the 5-FU microbeads maintained a relatively constant percentage swelling index from 30 to 90 min in the SGF, but demonstrated a notably higher percentage swelling index from 60 to 90 min in the SIF. This behavior can be attributed to the exchange of calcium ions from the microbeads in alkaline pH.

Furthermore, in the SIF, the 5-FU microbeads began to erode and undergo dissolution between 100 and 110 min. By 120 min, all 5-FU microbeads had completely eroded. They initially expanded rapidly upon immersion in the SIF, followed by a gradual dissolution towards the end. Ultimately, the 5-FU microbeads dissolved entirely by the 120-minute mark in the SIF, whereas in the SGF, the microbeads remained unchanged or experienced slight swelling. Conversely, the 5-FU microbeads maintained their shape in the SGF.

### 2.8 In vitro drug release

**Fig. 8:**
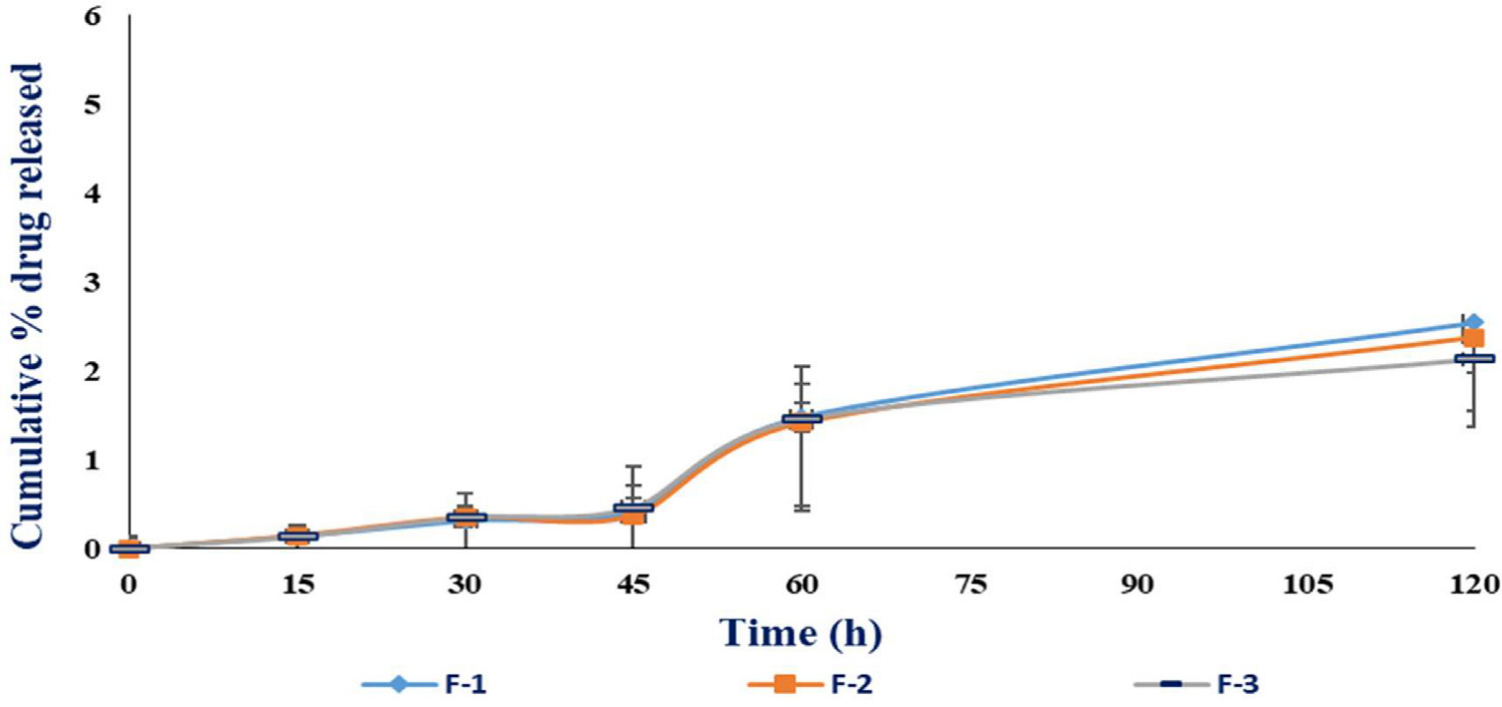
Cumulative percentage drug released of 5-FU microbeads in SGF (Mean ± SD, *n* = 3).

**Fig. 9:**
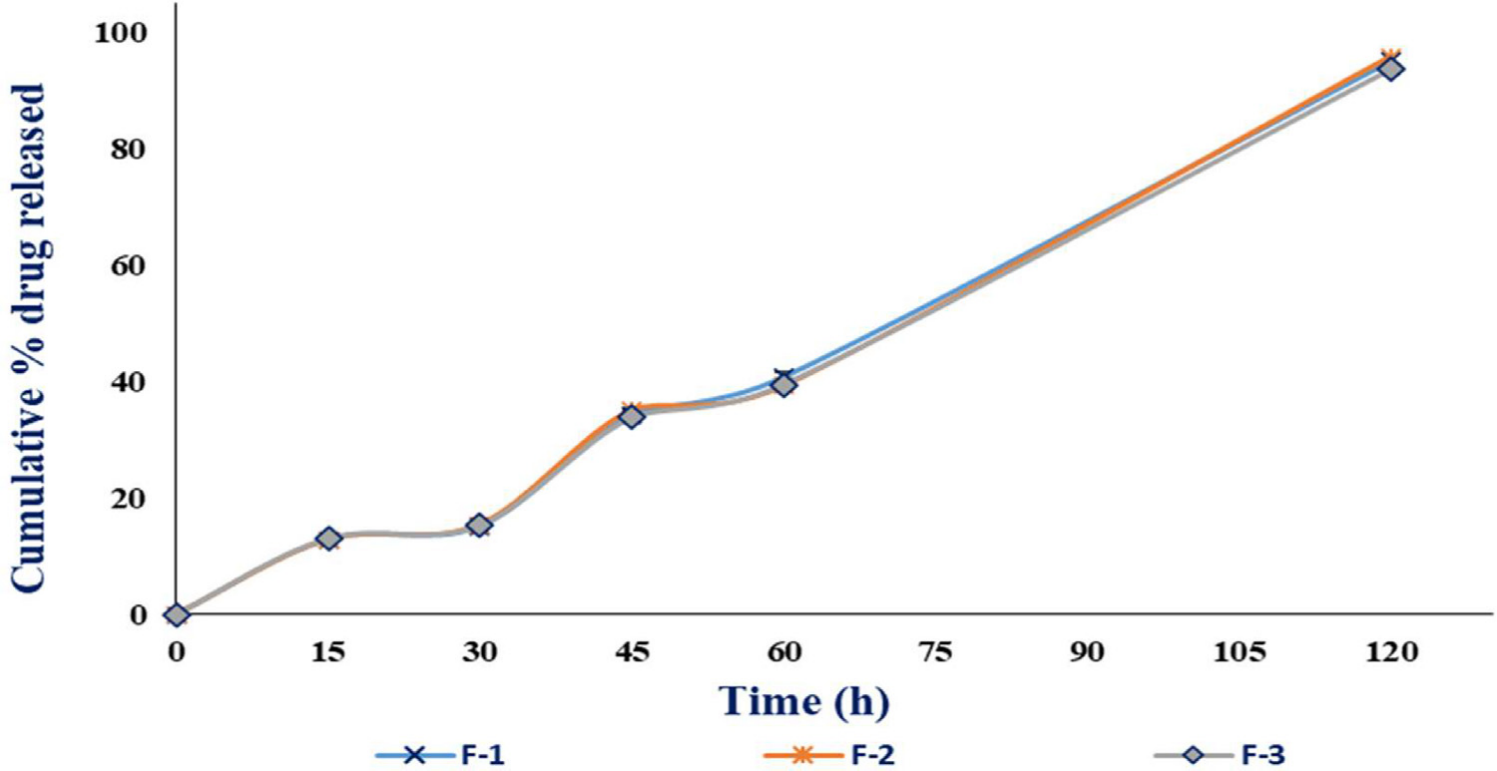
Cumulative percentage drug release of 5-FU microbeads in SIF (Mean ± SD, *n* = 3).

The drug release properties of the alginate microbeads are dependent on the swelling and dissolution of the microbeads. When the dissolution medium enters the alginate microbeads as the microbeads swell, the microbeads eventually started to undergo dissolution and the encapsulated active ingredient, in this case, 5-FU was released. The 5-FU microbeads drug release patterns are shown in Figs. 8 and 9.

The data shows that the 5-FU microbeads cumulative percentage drug release is higher in SIF, which is consistent with the data in swelling characteristics of the 5-FU microbeads which shows higher percentage swelling index in the SIF. This condition may be due to carboxyl group in the alginate microbeads that exhibit negative charge, causing electrostatic resolution to occur [6,7]. The cumulative percentage drug release of the 5-FU microbeads is less than 3% in SGF while it is more than 90% in SIF at 120 min. This data supports the hypothesis that the 5-FU alginate microbeads are able to be used as a drug carrier for colon targeted drug delivery systems, in which it does not release the content in the acidic environment of the stomach but release its content in the alkaline environment of the intestine or colon.

In short, the cumulative percentage drug release data shows that the 5-FU alginate microbeads display the pH dependent release properties and support the hypothesis that it can be used for colon targeted drug delivery systems.

## 3 Experimental Design, Materials and Methods

### 3.1 Materials

Sodium alginate was purchased from Manugel DMB, ISP, (Sigma-Aldrich, St. Louis, MO, USA). The characteristics of the sodium alginate of medium viscosity as well as high guluronic acid content was chosen. Another material, calcium chloride dihydrate was obtained from Sigma-Aldrich (Sigma-Aldrich, St. Louis, MO, USA). Meanwhile, 5-FU was obtained from Sigma-Aldrich (Sigma-Aldrich, Inc. St. Louis, MO, USA) [8].

### 3.2 Preparation of 5-Fluorouracil emulsion

The solution used for the encapsulation process was prepared by dispersing/ dissolving 5-FU (20 mg/ 20 mL) (9) in a mixture containing 1% w/v sodium alginate together with Tween 80 (1% w/v). (The nano emulsion was obtained by using ultrasonic homogeniser (QSonica, 53 Church Hill Rd. Newtown, PA, USA) for 55 s at 20% power) [9].

### 3.3 Characterisation of the alginate-5-fluorouracil emulsion

For the particle size distribution as well as the zeta potential, dilution of the emulsion was done before analysing. 0.1 mL of the emulsion, freshly prepared, was diluted with 9.9 mL of distilled water. Subsequently, the diluent was analysed by using a dynamic light scattering instrument (Malvern Zetasizer Nano series Nano-S and Nano-Z, Malvern Instruments Ltd., Worcestershire, UK) [10].

### 3.4 Preparation of alginate microbeads

The 5-FU alginate microbeads were prepared by using electrohydrodynamic atomization. The 5-FU emulsion was prepared with 1% of 5-FU together with 1% sodium alginate. As for the concentration of Tween 80, 1% w/v was chosen for the preparation of the microbeads. A syringe pump (Shenchen SPLab02, Baoding, China) was used to control the flow of the emulsion that is being loaded into a 10 mL plastic syringe, pumped through a 22 G needle. The needle tip was connected to a high voltage power supply (Analog technologies, Inc, San joes, CA, USA). Below the needle was the collector which would have a beaker containing a gelling bath, which was the 1% w/w calcium chloride. The needle was fixed at a distance of 10 cm above the collector. Meanwhile, the dripping rate and the voltage connected to the needle varied, with the dripping rate being 1 mL per min as well as the voltage being 7 kV. After that, the microbeads were kept in the calcium chloride solution for a period of 30 min together with continuous stirring to make sure that complete gelation has occurred.

After 30 min, the microbeads were removed from the calcium chloride solution and filtered by using a sieve. Ultrapure distilled water was used to wash the microbeads and they were left at the laboratory bench for drying. This process occurs at room temperature for a period of 16 h. Then, the following equation was used to calculate the percentage yield of the microbeads.

(1) $$\text{Beads}\;\text{yield}\;\left(\%\right)=\;\frac{\text{Amount}\;\text{of}\;\text{recovered}\;\text{beads}}{\text{Amount}\;\text{of}\;\text{the}\;\text{emulsion}\;\text{initially}\;\text{used}\;}\;x\;100\;$$

The blank beads, which are the alginate beads that do not contain the 5-FU, were prepared with the similar methods. The solution was prepared using 1% w/v sodium alginate together with Tween 80 (1% w/v) [2].

### 3.5 Microbeads characterisation

#### 3.5.1 Shape and size

A digital camera was used to capture the image of the dried microbeads. As for the shape and size, a Sigma ScanPro 5 image analyser was used for that purpose. At the same time, sphericity factor was used to determine the roundness of the microbeads [10]. The sphericity factor was shown as follow:

(2) $$SF\;=\frac{\left(\text{Dmax}\;-\;\text{Dper}\right)}{\left(\text{Dmax}\;+\;\text{Dper}\right)}$$

D_max_: Maximum diameter of the microbeads (mm)

D_per_: Diameter perpendicular to the Dmax (mm)

When the SF value is 0, it indicates a perfect sphere. Conversely, the higher the SF value, the more distortion is on the microbead shape. In this study, SF value of < 0.05 was taken as an indication of spherical microbeads.

#### 3.5.2 Encapsulation efficiency

The microbeads were disintegrated in buffered saline containing phosphate to transform the microbeads' polymer backbone into the emulsion phase. This process was conducted to quantify the amount of 5-FU encapsulated in the alginate beads. Subsequently, a UV–vis spectrophotometer (Shimadzu/UV-1700, Kyoto, Japan) was employed to measure the absorbance of the emulsion at 266 nm, indicating its turbidity. According to Alkhatib et al. (2018), their study demonstrated a correlation between the oil content and the turbidity of the emulsion [11]. A known amount of 5-FU was utilized to construct a standard curve. The encapsulation efficiency was calculated by using the following equation:

(3) $$\text{EE}\;\left(\%\right)=\;\frac{\text{Actual}\;\text{oil}\;\text{content}\;\text{in}\;\text{the}\;\text{microbeads}\;\left(\%\right)}{\text{Theoretical}\;\text{oil}\;\text{content}\;\text{in}\;\text{the}\;\text{microbeads}\;}\;x\;100$$

#### 3.5.3 Microbeads weight uniformity

An analytical balance was used to determine weight of the microbeads. In this process, 30 microbeads were chosen at random and weighed on the analytical balance [12]. Then, the weight of the 30 microbeads was taken. At the same time, the coefficient of the variation was calculated.

#### 3.5.4 Scanning electron microscopy

To observe the morphology and structure of the 5-FU loaded alginate microbeads, a Quanta 650 FEG scanning electron microscope (Hillsboro, Oregon, USA) was utilized. Prior to sputtering, the inner surface needed to be examined, so some dried microbeads were cut. For sputtering, aluminum stubs were used, and carbon adhesive tapes were employed for affixing the microbeads. The microbeads were then sputter-coated with carbon in thicknesses of 100 and 50, respectively, using a carbon sputter module. This process was carried out in a vacuum evaporator within an argon environment [13]. Finally, pictures of the coated microbeads were captured using an accelerating voltage of 5–10 kV under high vacuum conditions.

#### 3.5.5 Attenuated total reflectance-Fourier transform infrared spectroscopy

The ATR-FTIR spectra of the 5-FU containing alginate microbeads was recorded in the range of 4000–400 cm^−1^. This was done in an ambient air background in the resolution of 1 cm^−1^ (Nayak et al., 2014) by using PerkinElmer Spectrum 100 spectrophotometer (Perkin Elmer Corp., Norwalk, CT, USA). Then, the spectra were processed by using the SpectraGryph 1.2 spectroscopy software.

#### 3.5.6 Swelling characteristics

The weight gain of the microbeads in the aqueous media was measured to assess the swelling behavior of the 5-FU loaded alginate microbeads. Two types of media were used: simulated gastric fluid (SGF) with a pH of 1.2, simulating gastric conditions, and SIF with a pH of 6.8, simulating intestinal conditions. 0.1 g of microbeads was immersed in each of the two media for 2 h at a temperature of 37 °C, with a variation of 1 °C. Both media used were 50 mL each [13]. The swollen microbeads were removed from the media and filtered at predetermined time intervals, specifically every 10 min. To dry the swollen microbeads, a paper towel was used for this process. The percentage of swelling index was determined by using the following equation:

(4) $$\text{Swelling}\;\text{Index}=\;\frac{\left(\text{Wt}\;-\;\text{Wo}\right)}{\text{Wo}}\;x\;100$$

W_o_: Initial weight of dried beads

W_t_: Weight of swollen microbeads at t time

After measuring the weight of the microbeads, a digital camera was used to take the picture of the microbeads for the same time interval. Then, based on the morphology and thickness, the microbeads were characterised.

#### 3.5.7 In vitro drug release

An orbital incubator shaker (InnovaTM 4000 Benchtop Orbital Shakers, New Brunswick ScientificTM, Edison, NJ, USA) was employed for the dissolution of the microbeads. The process was conducted at a temperature of 37 °C with a variation of 1 °C, and a continuous stirrer set at 50 rpm. Initially, the dissolution tests were carried out with 200 mL of SGF (pH 1.2) by adding 0.1 g of the 5-FU alginate microbeads into the solution for 2 h. Subsequently, the tests continued using SIF (pH 6.8) for another 2 h. To assess the release properties of the microbeads, aliquots were collected, and a UV–vis spectrophotometer (Shimadzu/UV-1700, Kyoto, Japan) was utilized at 600 nm to measure the absorbance of the aliquot. This process involved extracting 5 mL of the aliquot from the solution and replacing the extracted amount by refilling the solution with 5 mL of new dissolution medium. The extraction process was carried out at regular 15-minute intervals [2].

### 3.6 Statistical analysis

For the statistical optimization, 2 software programs were used for this purpose, namely Minitab and Design-Expert 8.0.6.1 software (Stat-Ease Inc., Minneapolis, MN, USA). Meanwhile, KinetDS 3.0 Rev. 2010 software was used to analyse the squared correlation coefficient. All data are presented in mean ± standard deviation and every measurement was carried out in triplicate (*n* = 3). Data was tested for significant difference by using t-test ANOVA. P value of < 0.05 was used as the indicator that the data is significant as mentioned previously [2].

## Limitations

The in vitro and the in vivo characterisation such as the mucoadhesive test, biodistribution as well as the in vitro anticancer test were not carried out due to the lack of lab facilities unavailability at MAHSA University. Therefore, this data focuses on the preparation and characterisation of the 5-FU microbeads.

## Data Availability

Dataset of microstructure features of electro-hydrodynamic assisted alginate microbeads (Original data) (Mendeley Data)
